# Anticancer Activity of *Rosmarinus officinalis* L.: Mechanisms of Action and Therapeutic Potentials

**DOI:** 10.3390/nu12061739

**Published:** 2020-06-10

**Authors:** Alessandro Allegra, Alessandro Tonacci, Giovanni Pioggia, Caterina Musolino, Sebastiano Gangemi

**Affiliations:** 1 Division of Haematology, Department of Human Pathology in Adulthood and Childhood “Gaetano Barresi”, University of Messina, 98125 Messina, Italy; cmusolino@unime.it; 2 Clinical Physiology Institute, National Research Council of Italy (IFC-CNR), 56124 Pisa, Italy; atonacci@ifc.cnr.it; 3 Institute for Biomedical Research and Innovation (IRIB), National Research Council of Italy (CNR), 98164 Messina, Italy; giovanni.pioggia@cnr.it; 4 School and Operative Unit of Allergy and Clinical Immunology, Department of Clinical and Experimental Medicine, University of Messina, 98125 Messina, Italy; gangemis@unime.it

**Keywords:** *Rosmarinus officinalis* L., rosemary, carnosic acid, carnosol, cancer, leukemia, chemoresistance, synergistic effect

## Abstract

Alternative treatments for neoplastic diseases with new drugs are necessary because the clinical effectiveness of chemotherapy is often reduced by collateral effects. Several natural substances of plant origin have been demonstrated to be successful in the prevention and treatment of numerous tumors. *Rosmarinus officinalis* L. is a herb that is cultivated in diverse areas of the world. There is increasing attention being directed towards the pharmaceutical capacities of rosemary, utilized for its anti-inflammatory, anti-infective or anticancer action. The antitumor effect of rosemary has been related to diverse mechanisms, such as the antioxidant effect, antiangiogenic properties, epigenetic actions, regulation of the immune response and anti-inflammatory response, modification of specific metabolic pathways, and increased expression of onco-suppressor genes. In this review, we aim to report the results of preclinical studies dealing with the anticancer effects of rosemary, the molecular mechanisms related to these actions, and the interactions between rosemary and anticancer drugs. The prospect of utilizing rosemary as an agent in the treatment of different neoplastic diseases is discussed. However, although the use of rosemary in the therapy of neoplasms constitutes a fascinating field of study, large and controlled studies must be conducted to definitively clarify the real impact of this substance in clinical practice.

## 1. Introduction

General Consideration of Rosmarinus officinalis L.

Herbs and plants are a source of substances with possible anticancer action, which can block the process of carcinogenesis at different levels. Such molecules exhibit multiple abilities that affect diverse pathways by controlling the activity of several transcription factors [1].

*Rosmarinus officinalis* L., generally known as rosemary, is the accepted name (http://ipni.org/urn:lsid:ipni.org:names:455509-1) of a plant that originates in the Mediterranean area, and is cultivated in several countries [2]. Rosemary leaves are generally employed as flavoring elements and spices. Recently, rosemary extracts (REs) have been approved through European Union legislation, permitting food corporations to utilize the label, ‘‘antioxidant: rosemary extract’’ [3].

There is also growing interest in the pharmaceutical abilities of rosemary, employed in traditional medicine to enhance memory and alleviate pain, and for its anti-infective, anti-inflammatory and anticancer activity [4,5,6,7,8,9,10,11,12].

The active components of this herb are phenolic diterpenes and triterpenes. Phenolic elements are characterized by a chemical configuration of hydroxylated aromatic rings. They are generally present as secondary metabolites, and are the elements provided in the greatest quantities by food [13].

An analysis of all these substances suggests that diterpenes are the most effective molecules against neoplastic growth, followed by triterpenoids, which have demonstrated minor effectiveness. Numerous methods have been employed to attain an increased quantity of bioactive elements from rosemary, including liquid and supercritical fluid extractions (SFEs). Between them, SFE has been suggested as one of the most effective methods for obtaining the bioactive elements of rosemary [14].

Different types of analyses have been used to study the effects of these extracts on health. Among these effects, the anticancer action of RE and its main polyphenols (carnosic acid, carnosol, rosmarinic acid, rosmanol, methyl carnosate and betulinic acid, among others) has been well studied in in vitro (Table 1) and in vivo studies (Table 2), as outlined in the following sections.

## 2. Rosemary and Cancer: Mechanisms of Action

The anticancer activity of rosemary and its main derivatives has been correlated with diverse actions, including antioxidant effects, antiangiogenic properties, epigenetic action, the regulation of immune response and anti-inflammatory response, alteration of hormone signaling, modification of specific metabolic pathways and increased expression of onco-suppressor genes.

Rosemary has been primarily recognized as a possible anticancer drug mainly for its antioxidant activity. In fact, it has the capacity to act on free radicals, and could defend against their oxidative damage of DNA, proteins, and lipids [15], although, as subsequently observed, the derivatives of rosemary are, in some conditions, capable of inducing a cytotoxic effect precisely through the release of reactive oxygen species (ROS).

Besides its scavenging action, RE has also been stated to control intracellular antioxidant systems, by stimulating the activation of nuclear transcription factor (Nrf)2 target genes [16] and augmenting the glutathione level, with an increase in its reduced form (GSH) compared with that of its oxidized form (GSSG) [17]. However, because some antioxidants, such as beta-carotene, vitamin E and vitamin C, have given rise to controversial findings in clinical studies regarding their role in regulating the risk of cancer development [18,19,20,21,22,23,24], the correlation between the antioxidant effect and anticancer activity has been questioned.

Moreover, Carnosic Acid (CS) and Carnosol (CA) block some angiogenic functions of endothelial cells, such as differentiation, proliferation, migration and differentiation capacity. Several findings suggest that their effects on endothelial and cancer cell growth could be due to the stimulation of programmed cell death. Inhibition of in vitro angiogenesis by rosemary derivatives was confirmed by in vivo studies that employed the chick chorioallantoic membrane assay [25].

Moreover, CA blocks cytokine-induced adhesion molecule expression and monocyte adhesion to endothelial cells, via a system that involves NF-kB [26,27].

Rosemary and its derivatives are also known to have epigenetic action. Histone deacetylases (HDACs)—enzymes that regulate gene expression by acting on the acetyl group of histone—present anomalous expression that corresponds to the onset of tumors [28]. Notably, HDAC2 has been described to be highly expressed in tumor cells, where it decreases the expression of p53, leading to a reduction of programmed cell death.

Recently, authors examined the effect of RA with respect to suberoylanilide hydroxamic acid (SAHA), an HDAC inhibitor used as an antitumoral treatment, on the survival and programmed cell death of tumor cell lines and HDAC production. Similar to the effects of SAHA, RA reduced cell growth and blocked cancer spheroid formation, caused the apoptosis of tumor cells, and blocked the expression of HDAC2. Furthermore, RA reduced cyclins D1 and E1 and proliferating cell nuclear antigens, while p21 was increased. Finally, RA regulated the protein production of intrinsic mitochondrial apoptotic pathway-related genes, such as caspase-3, Bax, Bcl-2 and poly (ADP-ribose) polymerase 1 (PARP-1) (cleaved), through an increase in p53 derived from the HDAC2 decrease [29].

However, the antineoplastic action of rosemary could also take place through a regulatory effect on the immune system. Gomez de Cedron et al. examined the effects of a nutritional addition consisting of a supercritical extract of rosemary and shark liver oil-rich alkylglycerols (AKGs), as a possible complementary treatment for cancer subjects. Their findings indicate a positive effect on the immune system by means of stimulation of the innate immune response; this increased response is due to cytotoxic natural killer cells and the creation of an anti-inflammatory cytokine profile, which might support the immune response to cancer cells [30].

In a different study, the action of CS was evaluated in an experimental Balb/c mouse model of fibrosarcoma. CS was intraperitoneally dispensed daily to tumor-bearing mice for 7 days, and the effects were compared with two different groups: a group treated with cyclophosphamide and a group receiving vehicle only. CS significantly reduced tumor growth and promoted the reduction of splenic and tumor-associated Treg cells. CS also led to a reduction in splenocyte delivery of IL-4 and IL-10, and an increase in Interferon (IFN) production. However, CS did not elicit modifications in the ratios of CD4+ or CD8+ lymphocytes in the spleen, or in tumor-associated lymphocyte cells [31].

Alongside those reported above, further molecular mechanisms of rosemary have been described and related to its antitumor actions, including the modification of hormone signaling [32] and the ability to interact with a wide spectrum of molecular targets [33,34]. Moreover, it has been recently reported that rosemary is able to increase the expression of genes with established cancer-suppressing properties [35].

Finally, rosemary phenolic compounds may be involved not only in basic cellular processes or macro- and micro-nutrient metabolisms, but also in certain metabolic pathways. These modulated pathways could have a clinical influence on cancer onset and progression [36,37].

## 3. Rosemary: Usefulness of Treatment in Various Cancer Types

In the next paragraphs, we aim to report the most significant data from the literature related to the possible use of rosemary derivatives in the treatment of various neoplastic diseases.

### 3.1. Colon Cancer

Colon cancer is the third most common tumor diagnosed in the USA. According to the WHO, in 2018, 1.80 million new cases of colon cancer were identified, and 862,000 subjects died from the disease [38].

The effects of rosemary derivatives on this type of neoplasm have been highlighted through studies carried out in vitro, and on experimental animal models in vivo.

CA treatment considerably decreased the survival of human colon cancer HT-29, HCT116 and SW480 cells. In HCT116 cells, CA stimulated programmed cell death, which was due to the following mechanisms: stimulation of p53 and Bax; the reduction of Bcl-2, Mdm2 and Bcl-xl expression; and the stimulation of caspase-3 and -9. CA blocked the reporter gene activity of STAT3 in HCT116 cells by inhibiting the phosphorylation of JAK2 and Src kinases. Moreover, CA reduced the expression of STAT3 target gene molecules, such as cyclin D1, D2, and D3, as well as survivin. Finally, CA treatment caused the production of ROS in these colon tumor cells.

Pre-treatment of cells with the ROS scavenger N-acetyl cysteine reduced the inhibitory action of CA on JAK2-STAT3/Src-STAT3 signaling, and saved cells from CA-caused programmed cell death by inhibiting the stimulation of p53 and the cleavage of caspase-3 in HCT116 cells. Nevertheless, L-buthionine-sulfoximine, an inhibitor of GSH synthesis, increased CA-caused ROS production, thereby increasing the apoptotic action of CA [39].

The powerful cytotoxic effect of rosemary derivative on a panel of human colon tumor cells, including a cancer cell line derived from a primary cancer, was also reported in another investigation [40]. Nevertheless, the results of a proteomic-wide analysis demonstrated that each diterpene modifies protein homeostasis via different systems. In colon tumor cells, CA treatment drives the production of proteins implicated in the unfolded protein response, mirroring endoplasmic reticulum (ER) stress, while CS blocks the chymotrypsin-like activity of the 20S proteasome [34]. A connection between Unfolded Protein Response (UPR), Nrf2 stimulation and degradative processes was highlighted in the response of HT-29 cells to high levels of supercritical rosemary extract (SC-RE). Rosemary polyphenols led to proteomic modifications, possibly stimulating adaptive responses and leading to a stress decrease. Autophagy acts as a pro-survival system when the stress is moderate. However, it is possible that, as stress reaches a threshold, the adaptive compensatory systems cannot manage the cellular damage, causing cell death [41]. SC-REs were also demonstrated to block anchorage-independent cell growth, proving their inhibition of cancer cell transformation [42,43].

Perez-Sanchez et al. explored the anticancer action of RE. Modifications of the cell cycle by RE were demonstrated, such as a reduction in the G0/G1 phase with an increase in the number of colon cancer cells in the G2/M phase. A mild but statistically significant reduction was evidenced for Bax and HTRA, two proapoptotic proteins. A larger reduction was demonstrated for survivin in HGUE-C-1 and SW480 cells. The action of survivin is relevant in tumor onset and progression, and it has been implicated in chemo- and radio-resistance. An increase in survivin is linked to a worsened prognosis in subjects with colorectal tumors. However, all these modifications in the cell cycle and apoptosis do not seem to completely explain the powerful antiproliferative ability. Several studies have established that necrosis is the principal death mechanism [44].

Finally, a different approach was adopted by Valdes et al. They analyzed the effects of CA and CS on colon tumor HT-29 cell growth through the Foodomics approach, demonstrating that CA causes the transcriptional stimulation of genes that produce detoxifying enzymes. Metabolomics evaluation highlighted that treatment with CA modified the intracellular levels of glutathione. Furthermore, the Foodomics analysis was relevant in determining the connection between reduced concentrations of N-acetylputrescine and its degradation pathway at the gene level [45].

As mentioned above, the positive effects exerted by rosemary on the onset and progression of colon cancer have also been verified in experimental models in vivo.

Administration of a diet including CS (0.1%) in min + mice, which develop spontaneous colon cancers, reduced the multiplicity of intestinal tumors [46]. This report also stated that CS decreased the phosphorylation of β-catenin, and augmented the amount of β-catenin and E-cadherin at the intestinal enterocyte membrane [46].

In a different study, a shotgun proteomic method was used to analyze the action of RE on xenograft tumor development and global protein modifications in vivo. The daily administration of RE decreased the growth of colorectal cancer in vivo, with the deregulation of 74 proteins. A bioinformatic study of these proteins suggested that the RE essentially modifies RNA post-transcriptional modification, amino acid metabolism functions and protein synthesis, and the inactivation of the oncogene MYC was observed [47].

However, other in vivo studies have indicated different possible anticancer mechanisms exerted by rosemary, such as an effect on the non-coding genetic material with a known role in the onset of neoplastic pathologies, and the possible therapeutic action of which is now beginning to be understood [48,49,50].

Indeed, the antitumor effect of rosemary was found to be related to the reduction of serum miR-15b, a possible marker for controlling the effect of rosemary [51]. Furthermore, oral administration of methanolic RE to colorectal rat models reduced the concentration of additional serum colorectal cancer biomarkers, including colon cancer-specific antigen-4 and carcinoembryonic antigen [52]. A different in vivo study, using HCT116 colon cancer xenografted athymic nude mice fed 100 mg/kg/day RE dissolved in olive oil for 4 weeks, confirmed a reduction of tumor size in treated animals with respect to controls [53].

### 3.2. Gastric Carcinoma/Esophageal Squamous Cell Carcinoma

In the field of neoplastic diseases of the digestive system, various studies have shown the effects of rosemary on gastric carcinoma and esophageal squamous cell carcinoma.

Gastric cancer is the fourth most common cancer, while esophageal cancer is one of the more fatal tumors, representing the eighth most common cancer in the world and constituting 4% of all tumors.

In vitro research demonstrated that the crude extract of *R. officinalis* fruit exerts significant cytotoxic action on gastric cancer cells (AGSs) and KYSE30 cell lines. It was also demonstrated that AGSs were more responsive to the cytotoxic action of RE than the esophageal squamous cell carcinoma cell line. The mechanism of the effect on cancer cells was evaluated through cell cycle analysis. The findings demonstrated that RE triggered a G2/M cell cycle blockade in gastrointestinal cancer cell lines [54].

Moreover, Sageone, a substance isolated from rosemary, caused programmed cell death in SNU-1 human gastric cancer cells, and increased the cytotoxicity of cisplatin in SNU-1 cells, which are generally resistant to cisplatin. In contrast to cisplatin, which increased the levels of phosphorylated Akt, Sageone decreased the expression of Akt. Together with a subtoxic dose of cisplatin, Sageone had synergistic effects on programmed cell death stimulation in SNU-1 cells. The combined administration increased cleaved caspase-3 and caspase-9. These results indicate that Sageone is a promising anticancer drug, effective against gastric cancer [55].

### 3.3. Pancreatic Cancer

Additionally, the antineoplastic effect of rosemary has been demonstrated in pathologies other than colon or gastric cancer, such as pancreatic cancer. This type of neoplasm is the seventh most common cause of cancer-related death. Unlike other tumors, the occurrence of pancreatic cancer is increasing, with little progress in improving the survival rate [56].

Gonzalez-Vallinas et al. evaluated the anticancer action of supercritical REs on pancreatic cancer and colon cells. The data on cell viability demonstrate that the cancer cells sensitive to RE, from less sensitive to more sensitive, include PANC-1 (pancreas), MIA-PaCa-2 (pancreas), SW620 (colon) and DLD-1 (colon). Indeed, colon cancer cells are more responsive than pancreatic cancer cells to the antiproliferative actions of the extracts. In terms of possible mechanisms, an increase in the metabolic-related gene GCNT3 and a decrease in its potential epigenetic modulator miR-15b are correlated with the anticancer action of rosemary. Moreover, a plasmatic miR-15b reduction was discovered after in vivo treatment with rosemary [51].

### 3.4. Hepatocellular Carcinoma

Hepatocellular carcinoma (HCC) is the most frequent primary liver cancer, and the third leading cause of cancer death [57].

Treatment of HepG2 liver cancer cells with RA reduced ochratoxin- and aflatoxin-mediated cell damage, ROS levels and caspase-3 stimulation [58], and it led to increased caspase-8, NFBIA, TNFSF9 and Jun mRNA, and reduced Bcl-2 mRNA levels [59]. In Hep-3B liver tumor cells, RA was found to reduce cell viability [30], while in a different study, treatment demonstrated no relevant modifications of cell viability, but an increase in MRP2 concentrations, Nrf2 nuclear translocation, ARE-luciferin activity, intracellular ATP levels and efflux of p-glycoprotein [60].

Similar results have also been reported in other liver cell lines. *R. officinalis* L. essential oil was revealed to have powerful cytotoxicity effects on Bel-7402 cells [61]. RE dose-dependently reduced cell viability with an IC50 of 22.88 µg/mL [62].

Encouraging results have also been achieved in experimental animal models. In a diethylnitrosamine (DEN)-caused liver cancer model in F344 rats, RE was administered for 5 days intragastrically, with an intraperitoneal injection of DEN on day 4, after which the animals were fed a normal diet for 3 weeks until hepatectomy. Analysis of liver tissue demonstrated that RE may have some protective antioxidant actions [63]. According to this result, the livers of Swiss mice were exposed to 6 Gy ionizing radiation (IR) once and were fed 1000 mg/kg RE. Compared with IR-exposed animals that were not RE fed, a deferred onset of IR-induced mortality and reduced increase in glycogen and protein concentrations were seen in the livers of animals exposed to IR and fed RE [64]. However, attention should be paid to the high levels (1000 mg/kg) employed, some of which were 10 times greater than those discovered to exert powerful antitumor actions in other studies.

### 3.5. Lung Cancer

The American Cancer Society reported about 234,030 new cases of lung cancer and 154,050 deaths from the disease in the USA in 2018 [65].

In this field, studies have also been conducted on both cell cultures and animal models.

For instance, Moore et al. investigated the action of RE on the growth and programmed cell death of human non-small cell lung cancer (NSCLC) cells, and its effects on signaling events.

RE blocked cell growth and decreased the survival of NSCLC adenocarcinoma A549 cells, while programmed cell death was increased. RE drastically decreased total and phosphorylated Akt, p70S6K and mTOR levels [66].

In another study, different cell lines were employed. In NCI-H82 and A549 lung tumor cell lines, RA reduced cell proliferation, which was correlated with diminished hCOX2 function, suggesting an anti-inflammatory action from RA [67].

Remarkably, a population-based study indicated that an overall risk decrease in lung tumor occurrence was correlated with subjects’ consumption of rosemary (OR = 0.66, 95% CI = 0.37–1.15) [68]. On the basis of these data, several investigations were also performed on experimental animal models. For example, utilizing 1–4 mg/kg RA for 20 days in Lewis lung carcinoma (LLC) xenografted mice led to reduced cancer diffusion [69].

Furthermore, LLC-bearing mice were treated with cisplatin via intraperitoneal injection. They were fed CA. The combination of CA and cisplatin led to considerably better antiproliferation effects and increased programmed cell death in LLC xenografts than cisplatin alone. In regard to the mechanisms of this synergic effect, analysis demonstrated that CA oral gavage improved the function of CD8+ T cells, as demonstrated by greater IFN-γ production and greater productions of perforin, granzyme B and FasL [70].

However, different mechanisms of action have also been considered. For instance, myeloid-derived suppressor cells (MDSCs), operating as one of the main immune-suppressive cells impeding the anticancer immune response, are cells that originate from the myeloid lineage and amass aberrantly in lymphoid tissues and blood in cancer subjects. MDSCs could not only block anticancer immune defenses but also increase cancer proliferation and diffusion [71].

In one study, the percentage of MDSCs in cancer tissues was decreased by CA administration. The use of CA decreased the mRNA levels of MMP9, iNOS2 and Arg-1, which are functional indicators for MDSC [70].

### 3.6. Cerebral Neoplasms

Cerebral neoplasms constitute a group of pathologies for which rosemary and its derivatives seem to represent a potential therapeutic resource.

In fact, a study was conducted to determine if CA has an antiproliferative effect on human glioblastoma (GBM) cells, and to verify the molecular mechanisms involved. The findings revealed that CA reduced the survival of GBM cells, but it also decreased the survival of normal astrocytes. However, in GBM cells, CA triggered early G2 inhibition, increased the expression of p21 WAF, decreased the fraction of cells presenting Ki67, and increased programmed cell death. Moreover, it was demonstrated that CA increased the proteasomal degradation of several proteins, including SOX2, retinoblastoma (RB), Cyclin B1 and glial fibrillary acid protein (GFAP), while MYC concentrations were unchanged, promoting a substantial alteration of cell cycle control [72]. However, a different study performed on T98G glioblastoma cells allowed for the definition of diverse antitumor mechanisms. CA stimulates the generation of Nerve Growth Factor (NGF), controlled by the Nrf2 signaling pathway [73,74]. Moreover, NGF is implicated in the control of proliferation and the survival of diverse target neurons; thus, it can act to defend neural cells from toxic elements that may promote tumors.

In IMR-32 neuroblastoma cells, CA caused an increase in programmed cell death by stimulating caspases and the p38 MAPK pathway, and reducing cell survival, which was correlated with reduced ERK activation [75]. However, interestingly, in SH-SY5Y neuroblastoma cells, CA reduced programmed cell death induced by the neurotoxic substances methylglyoxal and amyloid β, thus exerting a cytoprotective action on tumor cells [76,77]. This defense activity was correlated with the augmented stimulation of PI3K/Akt signaling, the blocking of cytochrome c release and the reduction of caspase cascades [76], whereas in U373MG astrocytoma cells, CA reduced amyloid β peptide generation and delivery, correlated with the stimulation of the α-secretase TACE/ADAM17 [78].

### 3.7. Ovarian Carcinoma/Cervical Cancer

Some positive effects of rosemary have also been identified in the neoplasms of the female reproductive system. For instance, essential oils of rosmarinus triggered a powerful reduction of cell growth in the human ovarian carcinoma cell line A2780, and an intense cytotoxic effect on other human ovarian cancer cell lines, such as SK-OV-3 and HO-8910 [61,79].

Good results have also been achieved in patients with cervical cancer. This kind of cancer is provoked by the Human Papilloma Virus, and it is the most preventable tumor via treatment with precancerous lesions and the employment of vaccines.

*Rosmarinus officinalis* essential oil effectively blocked the development of HeLa cells with an IC50 of 0.011 [80]. However, in a different study, these results were not confirmed [81].

### 3.8. Skin Cancers

Other investigations have also been performed to assess the actions of rosemary on skin cancers, among which melanoma is the most common type. It presents minimal response rates to standard treatment and is associated with a poor prognosis in almost all patients.

CS blocked the diffusion of highly metastatic mouse melanoma B16/F10 cells in an in vitro assay. Moreover, CS drastically reduced the tyrosine phosphorylation of extracellular signal-regulated kinase (ERK) 1/2, p38, JNK and AKT, and the inhibition of activation of transcription factors c-Jun and NF kappa-B [82].

In several other experimental models, including BEAC, HT-1080 and HUVEC cell lines, CA reduced cell viability, adhesion and diffusion, increased programmed cell death, and caused cell cycle arrest [25,83].

Different anticancer effects have been recognized in other studies. A 65% (v/v) hydroalcoholic RE preparation decreased, in a dose- and time-dependent manner, the growth of the human melanoma A375 cell line, generally highly resistant to chemotherapy drugs. A study of the cell cycle demonstrated that RE reduced cell growth via cytotoxic and cytostatic actions. Assessment of ROS generation and protein carbonylation suggested that the reduction of growth was not due to the pro-oxidant action of RE, but rather to multifactorial actions. In fact, a proteomic investigation was performed to explore the molecular targets involved, and demonstrated that RE treatment of melanoma cells caused a significant decrease in the concentrations of proteins that are essential for cellular homeostasis maintenance, the reduction of which can impede cellular functions by triggering ER stress [84].

Finally, exposure to ultraviolet B-light (UVB) increases the possibility of developing diverse types of skin tumors [85]. Therefore, the identification of substances able to act as chemopreventive drugs for UVB-caused skin tumor development is desirable, as these molecules are often less toxic than traditional drugs.

In this area, the advantageous actions of rosemary have been proved in in vivo studies. Topical treatment of CS prior to the administration of 12O-tetradecanoylphorbol-13-acetate (TPA) twice a week for 20 weeks considerably reduced the number of papillomas in dimethylbenz(o)anthracene (DMBA)-caused mouse skin cancer. This protective action of CS was probably due to its inhibitory effect on the TPA-caused stimulation of the ornithine decarboxylase enzyme, which is a marker of cancer promotion [10].

Tong et al. also studied the positive chemopreventive effect of CS, and its possible mechanism of action. Their findings demonstrated that CS could decrease a UVB-induced reactive ROS increase, and thus decrease DNA damage. It could also decrease the UVB-induced generation of cyclobutane pyrimidine dimers (CPD) in keratinocytes, via its capacity to absorb UVB radiation. CS decreases DNA damage by decreasing the production of CPD [86]. This specific decrease could be due to the 280 nm absorbance wavelength required for making cis-syn CPD, which coincides with the 284 nm absorbance peak of CS [14].

Finally, in a different study, the clinical effects of rosemary on DMBA-initiated and croton oil-promoted mouse skin cancers were analyzed. The RE action was examined by evaluating the latency time, cancer occurrence, cancer weight and diameter, as well as oxidative stress. The findings suggest that RE can protract the latency time and reduce cancer occurrence, cancer load and cancer size [87,88,89,90,91,92,93,94]. The amount of lipid peroxidation was drastically decreased in the blood and liver. Moreover, decreased concentrations of glutathione were re-established in animals administered RE. Therefore, at a dosage of 500 mg/kg body wt/mouse, the oral dispensation of RE demonstrated a significant protective action against two-stage skin tumorigenesis [95].

### 3.9. Oral Cancers

Although relevant advances have been achieved for oral cancer therapy with radiation treatment and chemotherapy, the overall 5-year survival percentage of oral cancer subjects remains at about 50%, and has not substantially improved in the last four decades [96]. Oral squamous cell carcinoma often has a bad outcome due to cancer local diffusion and lymph node metastasis.

Oral carcinogenesis is a complex process that relies on the accumulation of carcinogen-caused genetic modifications throughout carcinogen-treated tissues [97]. 7,12-Dimethylbenz(a)anthracene (DMBA)-induced oral carcinogenesis in golden Syrian hamsters is a common and well-known experimental model for analyzing molecular and histologic changes that occur in oral carcinogenesis.

In a study, oral cancers were generated in the animals’ buccal pouches by administering 0.5% DMBA three times a week for 14 weeks. In hamsters treated with DMBA, 100% well-differentiated squamous cell carcinoma formation was demonstrated. Animals that were administered CA by oral gavage at a dosage of 10 mg/kg bw and treated with DMBA entirely avoided cancer onset. Moreover, CA administration also modified alterations in the expression of several proliferative (c-fos, PCNA, and cyclin D1), apoptotic (caspase -3 and 9, Bcl-2, p53), inflammatory (COX-2 and NFkB) and angiogenic (VEGF) molecules in favor of blocking altered cell growth [98].

### 3.10. Kidney Cancer

Urinary tract cancers appear to be an adequate target for rosemary derivatives. In Caki, kidney cancer cells, CA stimulated programmed cell death via ROS-caused endoplasmic reticular stress. Moreover, CA triggered an increase in apoptotic markers such as ATF4, caspase3, and CHOP [99], while CA elicited an increase in TRAIL-mediated apoptosis in Caki cells, and in other types of renal cell lines (AHCN and A498), via a change in endoplasmic reticular stress-related proteins, such as Bcl-2, CHOP, c-FLIP, DR5, Bim, PUMA and CHOP [100]. Likewise, RE exerted a cytotoxic effect on urinary bladder carcinoma cells 5637.

### 3.11. Prostate cancer

In the context of these diseases, the neoplasm for which the most interesting results of in vitro and in vivo studies are available is certainly prostate cancer [101].

The anticancer actions of CS on prostate tumors have been explored. Experimental findings have reflected the anti-growth capacities of CS, which are evident in a dose- and time-dependent manner in PC3 cells, with observed IC50 values of 48.3, 39.2 and 34 μmol/L at 24, 48 and 72 h, respectively. CS induced a cell cycle arrest at the G2 phase of the cell cycle, along with an increase in several regulatory proteins, such as p21 and p27, and a reduction of cyclin-dependent kinase (cdk) proteins-2 and -6 and cyclin-A, -D1, and -D2 [102]. CS also blocked the PI3K/Akt pathway. At doses of 20 and 40 μM, CS demonstrated a relevant reduction in the protein expression of Akt at phosphorylation sites Thr-308 and Ser-473. Moreover, CS also stimulated the 5′adenosine monophosphate-activated protein kinase (AMPK) pathway. The levels of the AMPK beta-1 regulatory subunit were augmented by 365% in CS-treated cells. AMPK and its downstream molecule mTOR have been regarded as possible therapeutic targets for tumor therapy. Generally, the mTOR protein is increased in prostate cancer. CS can cause a reduction in phosphorylation of the mTOR protein, thus causing a block of prostate cancer proliferation in vitro [102].

The possible mechanisms by which REs induce tumor cell death could also include an increase in mitochondrial-dependent programmed cell death, as RE caused an increase in the proapoptotic protein Bax and a reduction of the antiapoptotic Bcl-2 proteins [103,104].

However, the most interesting aspect of the relation between rosemary and prostate cancer is the possibility that rosemary can intervene through hormonal receptors. The androgen receptor (AR) is a well-known target for prostate tumors. However, the advantages of antiandrogens are short term. Molecular changes happen rapidly within the AR, causing antiandrogen resistance in almost 50% of patients.

RE was examined to evaluate its capacity for increasing the degradation of the androgen receptor. Two prostate cancer cell lines (22Rv1 and LNCaP) and prostate epithelial cells from subjects submitted to prostatectomy were treated with RE. RE was able to reduce androgen receptor expression, which seems to be controlled by the expression of CHOP/GADD153 [32].

Moreover, diverse in vivo experiments have proved that REs block cellular growth in mice xenografted with prostate tumor cells. In these reports, the authors evaluated the concentrations of Sestrin 2, AR and prostate-specific antigen. They demonstrated that when RE was consumed orally, cancer proliferation was considerably reduced (by 46%) compared with animals not taking RE [32].

### 3.12. Breast Cancer

The advantage of REs and their derivatives in breast cancer prevention and therapy has been shown in diverse experiments. RE was able to reduce the survival of breast cancer cell lines [11,62], and CA was found to be the most efficacious element [105].

CS was reported to perform its antioxidant action via the estrogen receptor (ER) signaling pathway [106], and was discovered to be an antagonist of this receptor without any agonist action [107]. The influence of CS on cell growth, and its ER α and β’s specific control mechanisms in ER-positive breast cancer T47D cells, have been studied [108]. The findings demonstrated that CS exerts its inhibitory action on the growth of ER-positive breast cancer cells via target cell ER, mainly through the ERβ pathway. CS increased ERα and ERβ levels in T47D cells and the ERα/ERβ ratio. [108].

The action of CS on the target triple-negative breast cancer (TNBC) cell line MDA-MB-157 has also been explored. TNBC is believed to be an extremely aggressive type of tumor with a poor prognosis. Data from the study reported that CS inhibited the cell cycle at the G2 phase, and caused ROS-dependent programmed cell death and beciln1-independent autophagy in this type of breast cancer cell [109].

The role of ROS production in the induction of autophagy and cell death in several types of tumor cells has been demonstrated [110]. CS increased ROS generation in breast cancer cells in a dose- and time-dependent manner. It was observed that CS, at a non-cytotoxic level, caused a low increase in ROS production and triggered autophagy. Moreover, cells that were in contact with a greater quantity of CS for a short time also stimulated autophagy as a self-defense survival system. Nevertheless, protracted contact with high levels of CS caused disproportionate ROS generation, which induced apoptosis of breast cancer cells via the stimulation of intrinsic and extrinsic apoptotic pathways [110].

Gonzalez-Vallinas et al. studied the anticancer properties of supercritical fluid rosemary extract (SFRE) in different breast cancer cells, and highlighted that SFRE drastically increased the action of drugs such as tamoxifen, paclitaxel and trastuzumab [111].

In ER-negative human breast cancer cells, CA at a low dosage stimulated the expression of three genes implicated in glutathione synthesis (CYP4F3, GCLC) and transport (SLC7A11). At a greater dosage, CA stimulated the expression of other genes involved in antioxidant action (AKR1C2, TNXRD1, HMOX1) and apoptosis (GDF15, PHLDA1, DDIT3). At a high dose, CA reduced the expression of genes implicated in the inhibition of transcription (ID3) and the cell cycle (CDKN2C) [112].

The positive effects exerted in vitro on breast cancer cells have also been confirmed by in vivo studies. Singletary et al. demonstrated that RE was able to reduce DMBA-caused mammary cancers in rats [113]. The administration of RE in the diet, at a dose of 0.5% w/w for 2 weeks, caused a significant reduction in DMBA–DNA adducts, while CS (at 1.0% w/w) did not demonstrate this action after oral administration. However, RE and CS blocked the formation of DMBA–DNA adducts and reduced cancer occurrence after administration via i.p. (200 mg/kg daily for 5 days) [113]. Furthermore, the introduction of methanolic RE at 2% w/w into the diet changed the metabolism of estrogens, increasing the liver metabolism of estradiol and estrone, and reduced their uterotropic action in female ovariectomized animals [114].

### 3.13. Hematological Neoplasies

The efficacy of rosemary and its derivatives has been demonstrated not only in the context of solid neoplasms, but also in hematological neoplastic pathologies.

A study performed on the leukemia HL-60 and K-562 human cell lines, and the murine RAW264.7 macrophage/monocyte cell line, demonstrated significant growth reduction, with an IC50 of 0.14% (1.4 mg/mL) and 0.25% (2.5 mg/mL) for HL-60 and K-562 cells, respectively. Moreover, RE considerably promoted the differentiation of HL-60 cells [11]. RE reduced survival in K-562 leukemia cells. Analogous actions of RE were described in other studies that reported the reduced growth of K-562 cells [115].

RA reduced cell survival, and reversed the induction of hyperosmosis-caused programmed cell death and associated ROS/ nitrogen (RNS) generation in K562 leukemia cells [116]. In U937 leukemia cells, RA increased TNF-α-induced apoptosis, and reduced TNF-α-caused NF-κB activation and ROS generation [117]. Unexpectedly, AKT1 and ERK2 concentrations, which control cell survival, were not modified by RA administration in U937 or K562 cell lines [115]. Moreover, RA promoted macrophage differentiation caused by all-trans-retinoic acid (ATRA), which was mediated by an increase in CD11b concentration on the cell surface [118]. In HL-60 leukemia cells, RA reduced cell proliferation, and this event was correlated with an increase in programmed cell death and with a reduction of dNTP concentrations [119]. RA treatment of other leukemia cell lines, such as CCRF-CEM and CEM/ADR5000, led to the development of enhanced cytotoxicity, necrosis, cell cycle arrest and blockage of p65 nuclear translocation [120,121].

One study indicated that CS caused programmed cell death in adult T-cell leukemia/lymphoma (ATL cells), a mortal malignancy triggered by infection with human T-lymphotropic virus type-1. A proteomic study reported that CS increased the levels of reductases, enzymes that have a central role in the glycolytic pathway, and enzymes in the pentose phosphate pathway. These enzymes are implicated in the generation of nicotinamide adenine dinucleotide phosphate (NADPH) [122]. The programmed cell death-stimulating action of CS may be correlated with NADPH-dependent redox control in the cells [27]. Therefore, this action of CS in ATL cells might be correlated with a reduction of glutathione [123].

In addition to the effect of rosemary on cultures of acute leukemia cell lines, there are data in the literature about the action of rosemary on chronic myeloid leukemia cell lines (CMLs).

The CML KBM-7 cell line was employed in a study, and findings suggested that CA has a considerable antitumor effect on CML KBM-7 cells with an IC50 of 25 µM. The antiproliferative action was related to the stimulation of programmed cell death and cell cycle arrest. Moreover, it was reported that the blocking of CML KBM-7 cell growth after CA administration could essentially be due to a decrease in microRNA-780 expression [124].

These findings suggest that CA could be a useful substance in the treatment of CML [124].

## 4. The Effects of Rosemary on the Metastasis Process

In order to spread, epithelial tumor cells need to infiltrate through the basement membrane and disrupt the extracellular matrix (ECM). In this regard, proteases have a central action, as they can alter the ECM elements, and thus favor tumor cell diffusion. It is a widely recognized fact that cancer cells generate greater quantities of proteolytic enzymes than normal cells. The matrix metalloproteinases (MMPs) MMP-2 and MMP-9, and the urokinase plasminogen activator (uPA), can degrade several ECM constituents, and are promoters of cancer diffusion and metastasis [125]. As reported above, COX-2 is increased in human and murine cancer cell lines, and COX-derived prostaglandin (PG) can increase the delivery of MMPs and cancer cell diffusion, ultimately supporting metastatic dissemination [126].

Rosemary could also have a role in cancer progression by reducing the metastasization process. After 24 h, CA treatment inhibited migration of the Caco-2 cell line, probably by decreasing the action of proteases such as uPA and MMPs. These actions may be due to a mechanism implicated in the blocking of the COX-2 pathway, as it was evidenced that CA decreases the levels of COX-2 in Caco-2 cells. Furthermore, CA has an inhibitory effect on Caco-2 cell adhesion to fibronectin surfaces and type I collagen. Moreover, inhibition of the dissemination and pseudopodial extension of cells pre-treated with CA was observed [127].

## 5. Synergistic Action and Chemoadjuvant Effect of Rosemary

It is likely that chemoresistance is the main impediment to the effective and efficient therapy of neoplastic diseases. Rosemary and its derivatives have proven to be able to reduce the phenomenon of chemoresistance and to enhance the effect of chemotherapy.

The effects of dietary rosemary on the activity of the human drug efflux transporter P-glycoprotein (MDR1, ABCB1) and multidrug resistance protein 1 (MRP1, ABCC1) were explored by employing P-glycoprotein-overexpressing hMRP1 gene-transfected KB/MRP cells and human cancer KB-C2 cells. The accrual of daunorubicin, a substrate of P-glycoprotein, in KB-C2 cells increased in the presence of CA and CS. The ATPase actions of P-glycoprotein were increased by CA and CS. Moreover, KB-C2 cells were sensitized to vinblastine cytotoxicity by CA, indicating that CA inhibits multidrug resistance. These findings imply that rosemary may be beneficial in increasing the effectiveness of cancer chemotherapy [128].

These data were confirmed using various types of neoplastic cell lines and the most diverse chemotherapeutics. Plouzek et al. stated that methanolic RE, consisting of 7.5% w/w CA and 7.5% w/w CS, increased the intracellular accrual and blocked the efflux of vinblastine and doxorubicin—P-glycoprotein (P-gp) substrates—in breast tumor MCF-7 cells overexpressing this glycoprotein [129].

Moreover, 5-Fluorouracil (5-FU) is the most utilized drug in colorectal cancer. Nevertheless, resistance to this compound is quite common, and different therapeutic strategies are needed. The actions of SFRE were studied in diverse human colon cancer cell lines. Findings demonstrated that SFRE acts synergistically with 5-FU on colon cancer cells. Moreover, SFRE increased the sensitivity of 5-FU-resistant cells to the therapeutic effect of this drug, representing a beneficial agent against both 5-FU-sensitive and -resistant tumor cells. Gene expression analysis suggested that the increase in the efficacy of 5-FU by SFRE might be due to the decrease in TYMS and TK1, enzymes associated with 5-FU resistance [43].

Similar results have been obtained in glioblastoma cell lines. It was demonstrated that the administration of etoposide, RE, and RE combined with etoposide was efficacious in destroying GBM cells, and RE enhanced the cytotoxicity of etoposide. Moreover, RE by itself promoted the growth of Mouse Embryonic Fibroblast (MEF) cells, while reducing the negative impact of etoposide on these cells when employed simultaneously with etoposide. It can be presumed that RE protects healthy cells from the negative effects of etoposide, without reducing its action in GBM cells [130].

Some studies have been able to at least partially clarify the mechanisms of the action of rosemary on chemoresistance in this type of cell. In fact, it was observed that CS could sensitize U87MG cells to treatment through the direct activation of the p53 pathway. Even though these cells present wild-type p53, the operational action of this onco-suppressor protein is altered by increased levels of the physiological p53 inhibitor mouse double minute 2 (MDM2) [131]. CS reduced the growth of diverse GMB cells, especially cells that present wild-type p53. The results demonstrated that CS decreased the growth of GMB cell lines through the dissociation of p53 from its endogenous inhibitor 2MDM2, and increased intracellular p53 levels in GBM cells [132].

The joint administration of RE and cisplatin was also examined in both cisplatin-sensitive and -resistant ovarian cancer cells, and it resulted in synergistic action. The authors demonstrated that CA, CS and rosmarinic acid were able to synergize with cisplatin in drug-sensitive cells, but only CS demonstrated this action in drug-resistant cells, suggesting that the synergy differs between the two cell types [133].

Synergistic action has also been revealed in melanoma cell lines. Carmustine (BCNU) and lomustine (CCNU) are two nitrosoureas with alkylating activity. CA can increase BCNU- and CCNU-caused cytotoxicity, and trigger cell cycle arrest in B16F10 cells [134].

In preclinical studies, vitamin D and CA revealed synergistic proliferation-blocking actions in leukemia cell lines. Steiner et al. discovered that CA increased the antiproliferative and differentiating actions of 1,25-dihydroxyvitamin D3 in human myeloid leukemia cell lines HL60 and U937 [135]. As for the possible mechanisms, CA and 1,25-dihydroxyvitamin D3 synergistically stimulated the Raf-MEK-ERK-p90RSK MAPK cascade in HL60 cells [136]. Moreover, administration of CA caused a reduction in the intracellular concentrations of ROS [137]. The synergistic effect was confirmed by in vivo studies. Treatment of leukemia-bearing mice with combined administration of a vitamin D analog and RE caused the normalization of white blood cells and increased survival, with respect to untreated animals [138]. These data have also been reported by other authors. RE improved the ability of vitamin D to stimulate programmed cell death, and enhance differentiation of WEHI-3BD murine leukemic and human HL-60 leukemic cells [139].

CA has also been shown to exhibit synergy with curcumin, a natural substance with known antineoplastic capacities and possible synergistic action with various chemotherapeutics [140,141].

In KO and HL-60 human Acute Myeloid Leukemia (AML) cells, combined administration of curcumin and CA provoked a synergistic growth-inhibitory action and a significant increase in programmed cell death, via both the extrinsic and intrinsic pathways. However, it is important to emphasize that these substances did not alter the survival of normal human fibroblasts, or that of proliferating and nonproliferating blood cells [142].

## 6. Conclusions

Alternative treatments with novel drugs are required for neoplastic diseases, as the effectiveness of standard therapy is often decreased by side effects. Several natural products have been demonstrated to be efficacious in the prevention and treatment of several types of tumors.

**Figure 1:**
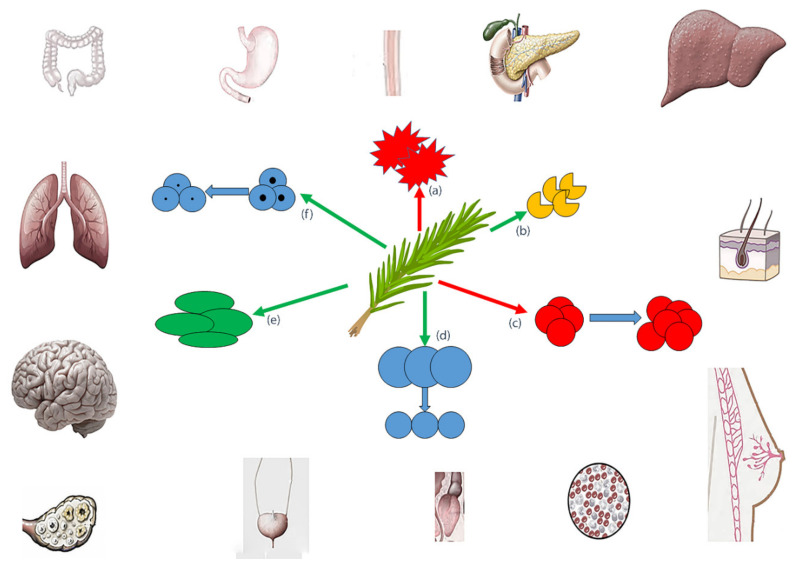
Rosemary effects on various types of cancers (red arrows: inhibition/blockade; green arrows: promotion): (**a**) stopping the activation of carcinogens, (**b**) increasing antioxidant enzyme activities, (**c**) reducing tumour-stimulating inflammation, (**d**) decreasing cell growth, (**e**) stimulating programmed cell death, (**f**) promoting the reduction of tumour angiogenesis and invasion.

Rosemary has been demonstrated to produce its actions by stopping the activation of carcinogens, increasing antioxidant enzyme activities, reducing tumor-stimulating inflammation, decreasing cell growth, stimulating programmed cell death, and suppressing tumor angiogenesis and invasion (Figure 1) [143].

However, we are probably still far from being able to use rosemary and its derivatives in clinical practice, and a significant number of problems have yet to be resolved.

First, it is essential to standardize the extraction system, in order to attain REs that always show the same antiproliferative capabilities. Some authors have proven that SFE REs have greater antiproliferative action with respect to aqueous and methanolic extracts in tumor cells [144,145].

A different problem is the difficulty in comparing different studies present in the literature. In fact, the effects of rosemary have been studied in many cancer cell lines, but the concentrations used in in vitro experimentations are widely variable. This high variability leads to the need for a more systematic analysis to detect efficient RE doses in vivo [146].

Another essential matter that must be analytically studied is the possible toxicity of the chronic administration of RE [69,147,148]. The cytotoxicity of RE in normal fibroblasts raises questions about its potential negative effects.

Irrespective of this, from the above information on the possible synergistic effects exerted by rosemary derivatives on most chemotherapeutic agents, the possibility should not be excluded that, in some cases, rosemary may negatively interfere with chemotherapy treatment [149]. For example, available results suggest that arsenic is non-enzymatically incorporated into NB4 cells, and establishes complexes that are dependent on intracellular GSH levels [150]. Moreover, arsenic complexes are known to be substrates of multidrug resistance proteins, and successively expelled. Thus, forthcoming research should concentrate on understanding the metabolite synthesis pathways of RA, by using genomic, transcriptomic, genomic and proteomic techniques. A molecular-level understanding of the regulatory elements of cells responsible for producing RA is essential for this aim. Moreover, a super-productive cell line should be established to achieve greater RA production. Furthermore, more efforts are necessary to create other plant or microbial species to biosynthesize RA heterologously [151].

In conclusion, although the use of rosemary and its derivatives in the therapy of neoplasms constitutes a fascinating field of study, large and controlled studies must be conducted to definitively clarify the real impact of this substance in clinical practice.

## Figures and Tables

**Table 1:** In vitro studies on the antineoplastic effects of Rosemary.

In Vitro Studies				
Disease	Cells	Rosemary Derivative	Mechanisms of Action	Ref.
**Colon Cancer**	HT-29, HCT 116, SW480	CA	Induction of apoptosis and inhibition of proliferation (increase of Bax; stimulation of caspase-3 -9; reduction of Bcl-2, Mdms, Bcl-xl; block of the reporter gene of STAT 3; inhibition of the phosphorylation of JAK2 and src kinases; reduced expression of cyclin D1, D2, D3, surviving).	[39]
	Tumour cells derived from a primary cancer	Rosemary derivative	Cytotoxic effect	[40]
	Colon cancer cell lines	CA	Stimulation of the unfolded protein response mirroring ER stress	[34]
	Colon cancer cell lines	CS	Block of chymotrypsin-like activity of the 20S proteasome	[34]
	HT 29 cells	Supercritical rosemary extract	Proteomic modifications (adaptive responses to decrease the stress)	[41]
	DLD-1, SW620	Supercritical rosemary extract	Block of anchorage-independent cell growth	[42,43]
	HGUE-C-1, SW480	RE	Reduction in G0/G1 phase; reduction of Bax, HTRA; reduction of survivin.	[44]
	HT-29 cells	CA, CS	Modification of glutathione levels, reduction of N-acetylputrescine.	[45]
**Gastric carcinoma, Oesophageal squamous cell carcinoma**	AGS, KYSE30 cell lines.	Crude extract of R. officinalis	G2/M cell cycle arrest	[54]
**Gastric carcinoma**	SNU-1 human gastric cancer cells	Sageone	Augmented phosphorylated Akt; increased cleaved caspase-3 and caspase-9	[55]
**Pancreatic cancer**	PANC-1, MIA-PaCa-2	Supercritical REs	Increase of the metabolic-related gene GCNT3, epigenetic action on miR-15b	[51]
**Hepatocellular carcinoma**	HepG2 liver cancer	RE	Reduced apoptosis, ochratoxin and aflatoxin-mediated cell damage, ROS levels and caspase 3 stimulation; increase in caspase 8, NFBIA, TNFSF9, Jun mRNA; reduction in Bcl-2 mRNA levels	[58,59]
	Hep-3B	RE	Augment in MRP2 concentrations, Nrf2 nuclear translocation, intracellular ATP levels	[60]
	Bel-7402 cells	R. officinalis L. essential oil	Increased cytotoxicity	[61]
**Lung cancer**	Human non-small cell lung A549 cancer cells	RE	Increased apoptosis; decreased total and phosphorylated Akt, p70S6K and mTOR levels	[66]
	NCI-H82 and A549	RA	Diminished hCOX2 function, anti-inflammatory action	[67]
**Cerebral neoplasms**	GMB cells	CA	Early G2 inhibition; increased expression of p21 WAF; increased apoptosis; increased proteasomal degradation of SOX2, retinoblastoma, cyclyn B1, glial fibrillary acid protein	[72]
	T98G GMB cells	CA	Increase of Nerve Growth Factor.	[73,74]
	IMR-32 neuroblastoma cell	CA	Increase of apoptosis by stimulation of caspases and p38 MAPK; reduced ERK activation.	[75]
**Ovarian cancer**	A2780, K-OV-3 and HO-8910 cell lines	Essential oils of rosmarinus	Reduction of cell growth; cytotoxic effect.	[61,79]
**Cervical cancer**	Hela cells	Rosmarinus officinalis essential oil	Reduced cell growth	[81]
**Skin Cancer**	Mouse melanoma B16/F10 cells	CS	Reduction of tyrosine phosphorylation of extracellular signal-regulated kinase (ERK) 1/2, p38, JNK, AKT and inhibition of activation of transcription factors c-Jun and NF kappa-B	[82]
	BEAC, HT-1080 cell lines	CA	Reduction of cell viability, cell adhesion and diffusion; increased apoptosis; cell cycle arrest	[25,83]
	Human melanoma A375 cell line	Hydroalcoholic RE	Cytotoxic and cytostatic actions; effect on ER stress	[84]
**Urinary tract cancer**	Caki kidney cancer cells	CA	Increased apoptosis via ROS-caused endoplasmic reticular stress.	[99]
	AHCN, A498, Caki kidney cancer cells	CA	Increase of TRAIL-mediated apoptosis	[100]
**Prostate cancer**	PC3 cells	CS	Cell cycle arrest at G2 phase; increase of p21 and p27; reduction of cyclin-dependent kinase proteins-2 and -6, cyclin-A, -D1, and -D2; block of PI3K/Akt pathway	[102]
	22Rv1, LNCaP cell lines	RE	Reduction of androgen receptor expression	[32]
**Breast cancer**	Breast cancer cell lines	RE, CS	Inhibitory action on the growth of Estrogen Receptor positive breast cancer cells via target ERβ pathway	[11,62,105,108]
	Triple-negative breast cancer cell lines MDA-MB-157	CS	Inhibition of cell cycle at the G2 phase; ROS-dependent apoptosis; beciln1-independent autophagy	[109]
	ER-negative human breast cancer cells	CA	Increased gene expression of CYP4F3, GCLC, SLC7A11, AKR1C2, TNXRD1, HMOX1, GDF15, PHLDA1, DDIT3. Reduced expression of ID3, CDKN2C	[112]
**Acute myeloid leukaemia**	Leukaemia HL-60 and K-562 cell lines	RE	Reduction of growth, augmented differentiation	[11,115]
	K-562	RA	Hyperosmosis-caused apoptosis; associated ROS/RNS generation	[116]
	U937 leukaemia cells	RA	Increased TNF-α-caused apoptosis; reduced TNF-α caused-NF-κB activation; ROS generation	[117]
	CCRF-CEM, CEM/ADR 5000 cell lines	RA	Augmented cytotoxicity, necrosis, cell cycle arrest and blockage of p65 nuclear translocation	[120,121]
**Adult T-cell leukaemia/lymphoma**	ATL cells	CS	Reduction of glutathione	[123]
**Chronic myeloid leukaemia**	CML KBM-7 cell line	CS	Stimulation of programmed cell death and cell cycle arrest; decrease of microRNA-780	[124]

**Table 2:** In vivo studies on the antineoplastic effects of Rosemary.

In Vivo Studies				
Disease	Experimental Model	Rosemary Derivative	Mechanism of Action	Ref.
**Colon cancer**	Min + mice	CS (0.1%)	Decreased phosphorylation of β-catenin; augmented amount of β-catenin and E-cadherin at the intestinal enterocyte membrane.	[46]
	Xenograft tumour model	RE	RNA post-transcriptional modification; alteration of the amino acid metabolism and protein synthesis; inactivation of the oncogene MYC.	[47]
	Colorectal rat models	Methanolic RE	Reduction of miR-15b	[51]
	HCT116 colon cancer xenografted athymic nude mice	RE	Reduction of tumour size	[53]
**Hepatocellular carcinoma**	Diethylnitrosamine -caused liver cancer model in F344 rats	RE	Antioxidant action	[63]
	Swiss mice	RE	Augment in glycogen and protein concentrations in livers	[64]
**Lung cancer**	Lewis lung carcinoma xenografted mice	RA		[69]
	Lewis lung carcinoma xenografted mice	CA and Cisplatin	Increased anti-proliferation effect and apoptosis; improved function of CD8+ T cells; increased IFN-γ, perforin, granzyme B and FasL, reduced myeloid-derived suppressor cells, reduced mRNA levels of MMP9, iNOS2, and Arg-1	[70]
**Skin cancer**	DMBA-caused mouse skin cancer	CS	Inhibitory effect on 12O -tetradecanoyl phorbol-13-acetate	[10]
	Ultraviolet B light-induced skin cancer	CS	Decrease UVB-provoked reactive ROS increase and DNA damage; reduction of cyclobutene pyrimidine dimers	[86]
	DMBA-caused mouse skin cancer	RE	Reduction of lipid peroxidation	[95]
**Oral cancer**	DMBA-provoked oral cancer, golden Syrian hamsters	CA	Alterated expression of c-fos, PCNA, and cyclin D1, apoptotic (caspase -3 and 9, Bcl-2, p53, COX-2, NFkB, VEGF	[98]
**Prostate cancer**	Mice xenograft model	RE	Reduced cancer proliferation	[32]
**Breast cancer**	DMBA-caused mammary cancers in rats	RE, CS	Reduction in DMBA–DNA adducts	[113]
